# Yeast gene CMR1/YDL156W is consistently co-expressed with genes participating in DNA-metabolic processes in a variety of stringent clustering experiments

**DOI:** 10.1098/rsif.2012.0990

**Published:** 2013-04-06

**Authors:** Basel Abu-Jamous, Rui Fa, David J. Roberts, Asoke K. Nandi

**Affiliations:** 1 Department of Electrical Engineering and Electronics, University of Liverpool, Brownlow Hill, Liverpool L69 3GJ, UK; 2 National Health Service Blood and Transplant, Oxford, UK; 3 University of Oxford, John Radcliffe Hospital, Oxford OX3 9UB, UK; 4 Department of Mathematical Information Technology, University of Jyväskylä, Jyväskylä, Finland

**Keywords:** CMR1/YDL156W, G1/S transition, DNA replication, DNA repair, binarization of consensus partition matrix

## Abstract

The binarization of consensus partition matrices (Bi-CoPaM) method has, among its unique features, the ability to perform ensemble clustering over the same set of genes from multiple microarray datasets by using various clustering methods in order to generate tunable tight clusters. Therefore, we have used the Bi-CoPaM method to the most synchronized 500 cell-cycle-regulated yeast genes from different microarray datasets to produce four tight, specific and exclusive clusters of co-expressed genes. We found 19 genes formed the tightest of the four clusters and this included the gene CMR1/YDL156W, which was an uncharacterized gene at the time of our investigations. Two very recent proteomic and biochemical studies have independently revealed many facets of CMR1 protein, although the precise functions of the protein remain to be elucidated. Our computational results complement these biological results and add more evidence to their recent findings of CMR1 as potentially participating in many of the DNA-metabolism processes such as replication, repair and transcription. Interestingly, our results demonstrate the close co-expressions of CMR1 and the replication protein A (RPA), the cohesion complex and the DNA polymerases *α*, *δ* and *ɛ*, as well as suggest functional relationships between CMR1 and the respective proteins. In addition, the analysis provides further substantial evidence that the expression of the CMR1 gene could be regulated by the MBF complex. In summary, the application of a novel analytic technique in large biological datasets has provided supporting evidence for a gene of previously unknown function, further hypotheses to test, and a more general demonstration of the value of sophisticated methods to explore new large datasets now so readily generated in biological experiments.

## 1. Introduction

The rate of data generation in biology has been much faster than that of developments in data analysis and integration [1]. The volume of the generated data have reached a level that cannot be readily interpreted without some form(s) of preprocessing by computational methods to produce comprehensible summaries or to highlight small useful subsets of data in order to formulate new hypotheses to target future research. Although the complete exploration and interpretation of this large amount of raw data has become an increasingly challenging issue, raw data generation has not stopped; indeed, the rate of accumulation of data is accelerating.

Gene clustering is one of many classes of *in silico* computational methods that have been applied to high-throughput datasets for their analysis. Gene clustering based on the genetic expression profiles measured by microarrays aims to group the given set of genes in a set of clusters such that those genes that belong to one cluster are relatively more co-expressed with each other, while not so co-expressed with the genes that are assigned to the other clusters. Many studies achieved this by using different clustering methods such as k-means [2], hierarchical clustering (HC) [3], self-organizing maps (SOMs) [4], self-organizing oscillator networks (SOONs) [5] and others.

Because different clustering methods generate different clustering results, ensemble clustering methods have been proposed to scrutinize the results of many individual clustering experiments over the same set of genes in order to produce one consensus result. Some of these ensemble clustering methods are relabelling and voting [6], co-association matrix-based methods [7], hypergraph-based methods [8] and others.

A new paradigm of clustering has been proposed recently through a new ensemble clustering method called *binarization of consensus partition matrix* (*Bi-CoPaM*) that relaxes conventional clustering constraints and allows any gene to be assigned exclusively to one cluster, assigned simultaneously to multiple clusters or unassigned from all the clusters [9,10]. This method can be tuned to generate clusters with different levels of tightness ranging from very tight clusters, while leaving most of the genes unassigned, to very wide and overlapping clusters passing through conventional complementary clusters. The aim of this new paradigm of clustering is to be able not only to restructure the raw biological data into a set of well-defined clusters, but also, in the case of generating tighter clusters, to shrink the size of these clusters to more comprehensible levels appropriate to the specific requirements of research.

One of the genes that have been extensively considered in our Bi-CoPaM analysis is the newly characterized yeast gene CMR1/YDL156W. Our experiment had begun before the availability of the newly revealed information about this gene in three recent studies that have investigated this gene [11–13]. Choi *et al.* [11] used biochemical methods to find that the CMR1 gene's product binds the DNA and may be involved in DNA-damage responses. Gilmore *et al.* [12] used integrative bioinformatics, quantitative proteomics and biochemical approaches to conclude that CMR1 is a member of the core histone network and is highly associated with the four histones as well as with many of the other proteins within the network. Sufficient evidence was also found to associate CMR1 with many cellular processes such as chromatin remodelling, transcription and DNA repair/replication [12]. Even more recently, Tkach *et al.* [13] have thoroughly investigated the genetic and physical interactions between CMR1 and DNA repair genes that are localized in nuclear foci to characterize a previously unknown DNA response pathway.

In this study, we aim to highlight the most tightly co-expressed cell-cycle genes by applying the Bi-CoPaM method over a set of cell-cycle genes from two relatively recent yeast microarray datasets that have a high sampling rate [14]. Moreover, we investigate the tightest of the generated clusters while putting more focus on the CMR1 gene and its relation with the rest of the genes in the clusters. We also aim to compare our *in silico* gene-expression-based clusters with other CMR1-containing subsets of genes recently obtained by biochemical approaches in order to generate testable hypotheses for the CMR1 gene and other genes of previously unknown or poorly known function.

## 2. Datasets and experimental procedures

We apply the novel ensemble clustering method *Bi-CoPaM* [9,10] over the 500 most periodic cell-cycle-regulated genes in budding yeast from two different microarray datasets. The aim was to exploit the benefits provided by the new paradigm of clustering proposed through the Bi-CoPaM method to derive four tight clusters of highly co-expressed genes to generate hypotheses for further functional or genomic research.

The considered datasets are described in §2.1, a description of the Bi-CoPaM method is presented in §2.2 and the set-up of the Bi-CoPaM experiment over the datasets is described in §2.3.

### 2.1. Datasets

Two microarray datasets were generated for the yeast *Saccharomyces cerevisiae* genome using the α-30 and α-38 synchronization techniques, respectively [14]. Each experiment captures the profiles for the genes over 2 h that cover two complete cell cycles. The number of time samples in each is 25 with 5 min intervals between consecutive samples.

These two datasets as well as three older datasets synchronized by alpha [15], cdc-15 [15] and cdc-28 [16] were combined in Pramila *et al*. [14] and used to order the genes according to their periodicity in the cell cycle. The average time of peak expression for the 1000 most periodic genes was calculated in that same study as a percentage of the time progress in the cell cycle, i.e. peaking at 0 per cent means peaking at the M/G1 transition point, peaking at 50 per cent means peaking in the middle of the cell cycle and peaking at 99 per cent means peaking at the very end of the M phase.

The subset of genes that we consider in this study includes the most periodic 500 genes of these 1000 genes. We consider their profiles from both the α-30 and α-38 microarray datasets provided in Pramila *et al*. [14]. Electronic supplementary material, S1 lists the names of these 500 genes, their peaking times as percentages of the cell cycle that has been provided by Pramila *et al.* [14], and their normalized log-ratio expression profiles from both datasets α-30 and α-38.

### 2.2. Binarization of consensus partition matrix

Clustering methods have been increasingly applied over microarray datasets in gene discovery research. However, most of the traditional clustering methods apply the constraint that each gene must be exclusively included in one and only one cluster in the results. In many gene studies, it would be useful to develop different formats of the clustering results by obtaining either tight clusters with a few genes while leaving many genes unassigned to any cluster, or obtaining wide clusters that overlap or allowing single genes to be simultaneously included in multiple clusters. The Bi-CoPaM method described in Abu-Jamous *et al*. [9] relaxes conventional clustering constraints by allowing these forms of clusters to be obtained.

**Figure 1.**
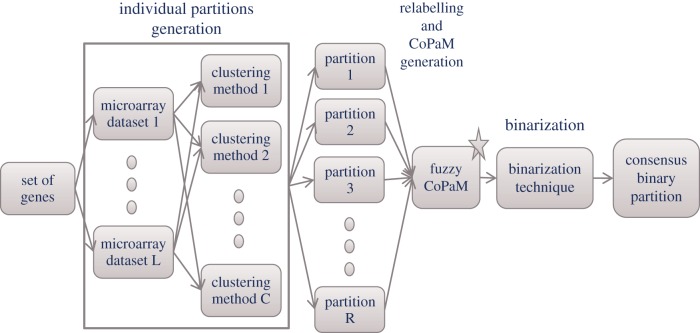
Flow chart summarizes the procedure followed by the Bi-CoPaM method. The first step is the generation of individual partitions by different clustering methods and based on multiple microarray datasets. These partitions are then relabelled and combined to produce a single fuzzy consensus partition matrix (CoPaM), which is then binarized to produce the final consensus binary partition [9,10]. (Online version in colour.)

The Bi-CoPaM method has four main steps illustrated in figure 1 and described in the following sections.

#### 2.2.1. Partitions generation

Different clustering results are obtained when different clustering methods are applied over the same set of genes either by using the same method with different parameters or the same stochastic method and parameters over different runs. Moreover, if the same method and parameters are used to cluster the same set of genes from different microarray datasets, then different results are obtained [9,17].

The first step of Bi-CoPaM is to produce *R* clustering results (partitions) by adopting *R* different clustering set-ups that vary in the adopted method, parameters and/or microarray dataset. These *R* partitions are represented by the partition matrices {**U**^1^, … ,**U***^R^*}, where each partition matrix has *K* rows representing the *K* clusters and *M* columns representing the *M* genes. The element 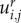 represents the membership of the *j*th gene in the *i*th cluster based on the *r*th partition.

#### 2.2.2. Relabelling

Because clustering is unsupervised, the *i*th cluster of one of the generated partitions does not necessarily correspond to the *i*th cluster of any other partition. The relabelling step aims to rearrange the clusters of the generated partitions, i.e. the rows of the partition matrices, such that they are aligned. After relabelling, the *i*th cluster of any partition will correspond to the *i*th cluster of each one of the other partitions. The problem of rearranging the rows of a partition matrix **U** to be aligned with a reference partition matrix **U**^ref^ is tackled by a min–max approach as follows [9,10]:

1. A dissimilarity matrix ***S****_K ×K_* is constructed such that its element **s***_i,j_* represents the dissimilarity between the *i*th cluster (row) of the partition **U** and the *j*th cluster (row) of the reference partition **U**^ref^.
2. The minimum of each column in ***S*** is calculated.
3. The maximum of these minima is located, then the clusters from **U** and **U**^ref^ that represent this maximum of minima are matched.
4. The row and the column in which that maximum of minima has been found are removed from ***S***.
5. Repeat the steps 2 to 4 until all clusters from both partitions are matched and the matrix ***S*** is empty.

The relabelled version of the partition matrix **U** is therefore denoted as 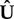, and the relabelling function is represented by 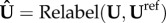.

#### 2.2.3. CoPaM generation

The relabelled partitions are scrutinized to generate a single fuzzy CoPaM that assigns each gene a different fuzzy membership value in each of the clusters based on the times in which this gene was included in the corresponding cluster in different individual clustering results. The membership values for any gene follow the constraints of fuzzy logic in that they can have any value between 0 (does not belong at all) to 1 (definitely belongs). The summation of the membership values of any gene in all of the clusters must be unity [9,18].

To generate the CoPaM, we define an intermediate CoPaM partition matrix **U**^int^ that initially represents the first partition **U**^1^. The rest of the *R* partitions are then fused within this intermediate partition matrix one by one. In each step, the next partition matrix to be fused into **U**^int^ is relabelled by considering this **U**^int^ as its relabelling reference. After all of the *R* partitions are fused, the final **U**^int^ represents the CoPaM U*. Let the intermediate partition matrix after fusing *k* partitions be **U**^int(*k*)^, then the mathematical formulation of this algorithm is

1. **U**^int(1)^ = **U**^1^.
2. For *k* = 2 to *R*
  (a) 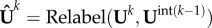  (b) 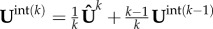
3. U* = U^int(*R*)^.

This final CoPaM U* is then passed to the final step, which is binarization.

#### 2.2.4. Binarization

Conventionally, the CoPaM is binarized; so that each gene is exclusively assigned to a single cluster and unassigned from all of the other clusters. Instead, Bi-CoPaM has the novelty in the binarization step that allows any gene to be either assigned exclusively to one cluster, or assigned simultaneously to multiple clusters, or unassigned from all of the clusters.

Six tunable binarization techniques have been proposed in the Bi-CoPaM method that can be used to generate tunable complementary, tight and wide clusters. Because we are mainly interested in tight clusters' cores rather than wide ones, in this paper, we would mainly adopt the *difference threshold binarization* (*DTB*) technique as well as the *maximum value binarization* (*MVB*) and *intersection binarization* (*IB*) techniques that are in fact special cases of the DTB. We describe these three techniques below and refer the reader to Abu-Jamous *et al*. [9] for the details of the other binarization techniques.

DTB assigns a gene to a cluster if the membership of this gene in that cluster is higher than its membership in all of the other clusters and is far from the closest competitor's membership with no less than a predefined parameter (**δ**). In other words, if a gene is not assigned to one cluster significantly more often than all of the others, it is considered a doubtful gene that has no certain cluster; thus, it would not be assigned to any of the clusters by this binarization technique. The higher the value of **δ** is, the tighter the clusters are and the larger the number of unassigned genes is. DTB is mathematically expressed as

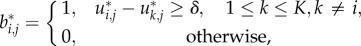

where 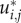 and 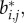 respectively, represent the consensus fuzzy and the consensus binary membership values for the *j*th gene in the *i*th cluster.

MVB assigns a gene to the cluster in which it has the maximum membership value whatever the difference from the competitor cluster is. This is equivalent to DTB with **δ** = 0 and does not leave any gene without being assigned to some cluster. IB assigns a gene to a cluster if all the individual clustering experiments assigned this gene to it consensually; it leaves the gene otherwise unassigned. This is equivalent to DTB with **δ** = 1.0 and generates the tightest possible clusters. MVB is mathematically expressed as

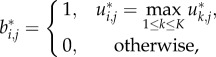



and IB is mathematically expressed as

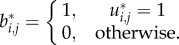



### 2.3. Experimental set-up

The profiles of the selected 500 genes from both α-30 and α-38 microarray datasets are clustered into four clusters by using the clustering methods: k-means [2], SOMs [4,19], HC [3] and SOONs [5,20]. Both bubble and Gaussian neighbourhood types are used in SOMs; complete, average and Ward's linkage techniques are used in HC; and varying values of three internal parameters are used in SOON. More about these clustering methods can be found in the relevant references.

The results of these individual clustering experiments are scrutinized to generate one fuzzy CoPaM that was then binarized by the DTB technique while varying the parameter **δ** from 0 to 1 in order to get varying levels of tightness for the clusters.

To justify our choice of clustering the 500 genes into four clusters, we have provided more detailed analysis in the electronic supplementary material, S2.

## 3. Results

### 3.1. Bi-CoPaM results

The numbers of genes (out of a possible 500) included in each of the four clusters C1, C2, C3 and C4 after applying the DTB technique with **δ** values from 0 to 1.0 are listed in table 1. The complete lists of genes included in each of the clusters at all of the considered tightness levels are included in the electronic supplementary material, S1. Note that DTB with **δ** = 0 is equivalent to MVB, and DTB with **δ** = 1.0 is equivalent to IB. It can be seen that with MVB, the total number of genes assigned to the four clusters is 500 which indicates that complementary clusters are generated where each gene is exclusively assigned to one and only one cluster. While increasing the value of **δ** to tighten the clusters, fewer genes are included in the clusters and more genes are left unassigned.

**Table 1.** Number of genes included in each of the four clusters at different *δ* values of the DTB technique. The shaded cases are the ones that are selected to be the clusters' cores.

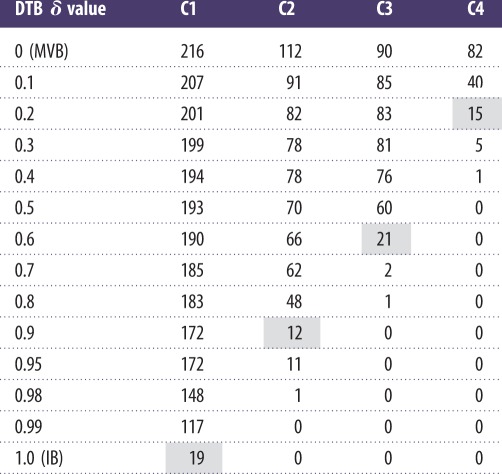

It can be seen that the cluster C1 is the tightest cluster as it is the only cluster to survive without being empty until IB. The rest of the clusters ordered by decreasing levels of tightness are C2, C3 and C4. Note that by moving from the absolute tightest case of C1 at IB with 19 genes to the case of DTB with **δ** = 0.95, which is indeed an extremely tight case, the C1 cluster inflates significantly to include 172 genes, whereas the other three clusters contain few genes if not empty. Less tight clusters derived with DTB and **δ** ≤ 0.95 do not show big differences in the numbers of genes included in C1.

To focus on a small subset of genes of potential importance, the smallest reasonable number of genes in each of the four clusters was chosen as the *core* of that cluster. The chosen cores' cases are shaded with grey in table 1. The cores' average peak times as percentages of the cell cycle as well as the expected corresponding cell-cycle phases from Pramila *et al*. [14] are listed in table 2. Based on the previous discussion, in the case of the C1 cluster, although the analysis concentrates on the core at IB, the genes down to DTB with **δ** = 0.95 are also considered significant and will be referred to as appropriate, see electronic supplementary material, S2 for more detail about the profiles of the genes included in C1 at these less tight levels. Revealing the difference in the precision of assignment for these four clusters as well as the ability of choosing different clusters' cores by tuning the level of strictness for different clusters are potentially useful outcomes of using the Bi-CoPaM method.

**Table 2.** For each of the four clusters' cores, the first to the fourth rows, respectively, show the average peak time as a percentage of the cell cycle, its standard deviation (s.d.), minimum (min.) and maximum (max.) values. The last row shows the estimated cell-cycle phase for each core based on its average peaking time.

cluster	C1 (%)	C2 (%)	C3 (%)	C4 (%)
average peak time for core genes	20	66	97	46
s.d.	3.2	3.3	4.9	6.7
min.	14	62	88^a^	40
max.	27	75	6^a^	67
expected cell-cycle phase	late G1/S	G2	M/early G1	S/G2

^a^These percentage values are cyclic, i.e. after 99%, the cycle goes back to 0%. So the earliest peak in C3 is at 88% of the cycle and the latest is at 6% of the next cycle.

**Figure 2.**
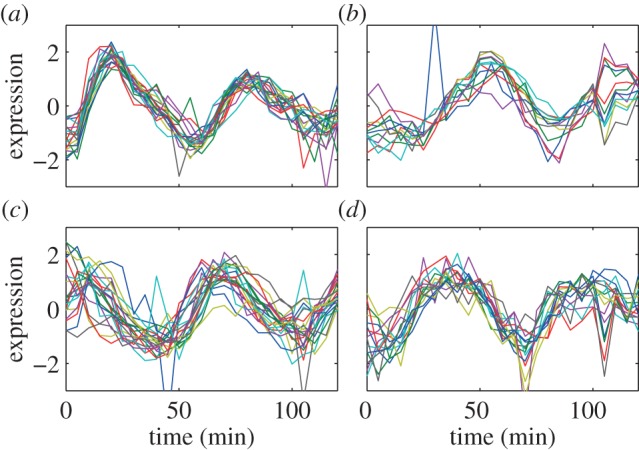
The expression profiles for the genes in the clusters' cores from the α-30 dataset. (*a*) C1–19 genes, (*b*) C2–12 genes, (*c*) C3–21 genes and (*d*) C4–15 genes. (Online version in colour.)

**Figure 3.**
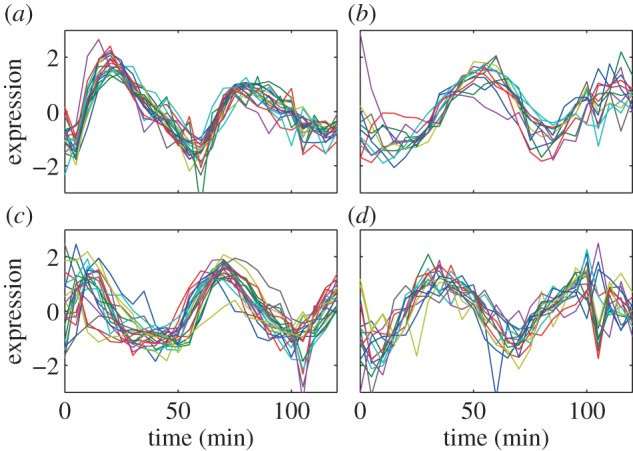
The expression profiles for the genes in the clusters' cores from the α-38 dataset. (*a*) C1–19, (*b*) C2–12 genes, (*c*) C3–21 genes and (*d*) C4–15 genes. (Online version in colour.)

The full lists of the genes in these four cores are listed in table 3, and the profiles for the genes in these core clusters from both α-38 and α-30 datasets are plotted in figures 2 and 3, respectively. Note that in the lists of genes, if any yeast ORF has not been characterized previously, and thus does not have a gene name, the ORF name is used instead.

**Table 3.** The names of the genes included in each of the four clusters' cores. For each of these clusters, the title row shows the cluster's label, the binarization configuration under which it has been chosen and the number of genes included in it.

C1 core at IB (DTB with **δ** = 1.0) (19 genes)		C2 core at DTB with **δ** = 0.9 (12 genes)	C3 core at DTB with **δ** = 0.6 (21 genes)		C4 core at DTB with **δ** = 0.2 (15 genes)	
AXL2	SLK19	BUD20	ASH1	PIG1	ABF1	YGL101W
CDC45	SMC1	CDC5	CHS1	PIL1	CSN9	YJL118W
CHR1	SMC3	CLB1	FAR1	PRY1	FLR1	YLR455W
CMR1	SPC42	CLB2	HSP150	PST1	GDA1	
EXO1	URH1	FET3	HXT2	ROD1	GDT1	
MSH2	YDL163W	FRK1	LSP1	SED1	MBP1	
POL2	YJR030C	PMP3	MCM2	TEC1	MSB1	
POL3		SCW4	MCM3	YLR194C	NDD1	
RAD27		SHE2	MCM4	YNL134C	SSA1	
RFA2		SML1	MCM5		STU2	
RNR1		SRC1	MCM7		TOF2	
RTT107		SWI5	NIS1		VID22

From figures 2 and 3, many observations can be made. First, the α-30 and the α-38 datasets have very close profiles except for some outlier values; this allows us to use either set for most of the remaining discussions. Second, the profiles of expression over time for the genes that are within each cluster's core clearly show the usefulness of the Bi-CoPaM approach in obtaining tighter and more specific clusters. Third, although all of these clusters' cores are tight, the cluster C1 is clearly the tightest, as shown by the **δ** value at which this core was obtained compared with the others (table 1). Finally, each set of genes in the four clusters' cores shows periodic peaking at a different stage of the cell cycle, which demonstrates clustering has derived sets of genes with distinct properties (table 2).

### 3.2. Gene ontologies analysis

We have performed *gene ontologies* (*GOs*) analysis for the genes included in the C1 cluster by using the GO Slim tool [21]. We have used this tool to search for biological processes, functions and components GO terms that are enriched in C1 at DTB with all of the values of **δ** reported in table 1. Full results lists of this GO Slim analysis for processes, functions and components have been provided in the electronic supplementary material, S3, S4, and S5, respectively.

## 4. Analysis and discussion

Our study is based on the computational analysis of high-throughput data from different experiments by using the Bi-CoPaM method, rather than by pure biological or biochemical experiments. It highlights important subsets of genes and has led to a set of proposed hypotheses. We hypothesize that CMR1 has a biological relationship with the replication factor A (RPA) complex, DNA polymerases **α**, **δ** and **ɛ** as well as the cohesion complex. Also our study provides support for some previously unconfirmed, or not fully understood, hypotheses such that the CMR1 gene is a potential target for the MBF transcription complex [22], that it has a role at the G1/S transition within the cell cycle [12], and that it has a role in DNA repair and some other DNA-related processes such as synthesis and transcription [11–13]. We also provide a set of novel clusters of genes with tunable co-expression levels that can serve as a resource for further focused gene discovery studies.

In reference [12], a quantitative proteomics approach was adopted to extend the protein network of core histones (H2A, H2B, H3 and H4) in the budding yeast *S. cerevisiae* and identified CMR1 as a member in this network. Some 556 proteins were found binding to one or more histones, whereas only 25 proteins of these were found binding to the four core histones. The 25 proteins include the four histones (H2A, H2B, H3 and H4), two units of the RPA complex (RFA2 and RFA3), two units of the Ku complex (YKU70 and YKU 80), many units of the RNA polymerase complex (RET1, RPO31, RPC17, RPC37, RPC40 and RPC82), many single-unit proteins (RIM1, YTA7, PSH1, CSE4, ABF2, CKA2, TIF3, DEM1, SUB2 and SMC3) and the previously uncharacterized protein YDL156W/CMR1. Then, associations with the CMR1 protein were investigated, and it was found that many proteins showed stable association with YDL156W, including the six proteins RIM1, RFA2, RFA3, YTA7, YKU70 and YKU80 which are within the 25 proteins found binding to all of the four core histones [12].

**Figure 4.**
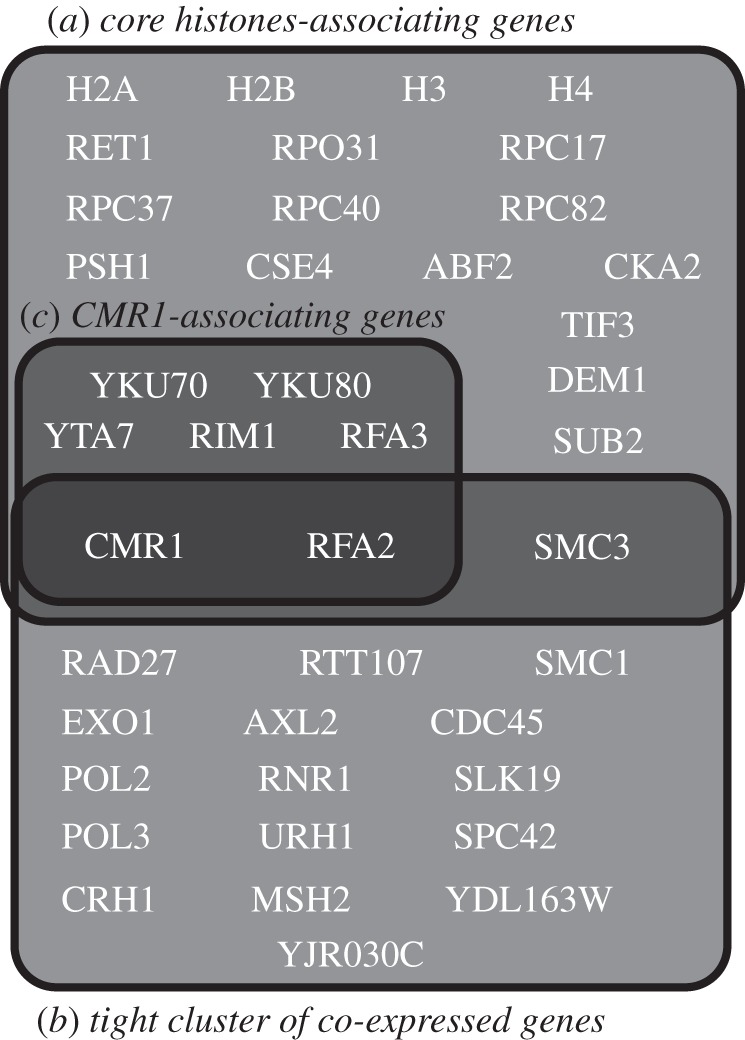
Venn diagram illustrating relations between the subsets of genes found by using quantitative proteomics to extend the core histone network and the subset of genes found by tight gene clustering based on gene expression profiles. The subset (*a*) represents the 25 genes found to be associated with the four core histones [12], the subset (*b*) represents the seven genes out of those 25 found to be associating with CMR1 [12] and the subset (*c*) represents the 19 co-expressed genes found in the tightest cluster of genes by using the Bi-CoPaM method in our study.

In our Bi-CoPaM gene expression analysis, CMR1 has been found in a small subset of 19 tightly co-expressed genes; figure 4 illustrates the relation between the core histone genes subset and our co-expressed genes subset. It can be seen that three of the 19 co-expressed genes, CMR1, RFA2 and SMC3, in Bi-CoPaM are found to be associated with all the four core histones. Moreover, RFA2 not only associates with the four histones, it associates with CMR1 itself and is co-expressed with it. Thus, Bi-CoPaM provides stronger evidence for the relation between CMR1 and RFA2 in the cellular processes.

It is worth mentioning that in our results the histones themselves have been found in the cluster C4 at DTB with **δ** = 0.2 and not in the cluster C1 which includes CMR1 (see electronic supplementary material, S1). This is because the transcription of histones occurs in the S phase in order to synthesize the chromosomes of the forthcoming daughter cells [14,23]; recall from table 2 that the C4 cluster peaks at the S/G2 phase. Despite that, histone proteins exist within the nucleus, packaging the DNA molecules, at all of the stages of the cell cycle. Thus, although the CMR1 gene has not been found co-expressed with the histones themselves, it has been found co-expressed with many genes whose products interact with the histones.

We will now focus our analysis and discussion around the genes within the very tight novel cluster of genes obtained at the strictest binarization technique, IB. When needed, we shall refer to some of the genes included in the C1 cluster at just below the tightest binarization levels such as DTB with **δ** values of 0.99, 0.98 and 0.95. The full gene membership lists for the four clusters at all of the cases listed in table 1 are provided in the electronic supplementary material, S1. They can serve as an important resource for further yeast gene discovery studies.

### 4.1. Replication factor A

RFA2 is part of the heterotrimeric RPA complex that also includes RFA1 and RFA3 proteins. RPA participates in many of the DNA-metabolism pathways such as DNA replication, recombination, repair and transcription [24]. In addition to Gilmore *et al*.'s [12] findings, Tkach *et al.* [13] also reported genetic and/or physical interactions between the CMR1 gene and all of the three components of the RPA complex. Interestingly, in our results at DTB with **δ** = 0.99, RFA1 is included within the cluster C1, whereas RFA3, to its slightly noisier profile, can be found in C1 by going back to complementary clusters at MVB. Thus, the three components of the RPA complex are seen to be clustered with CMR1 from the Bi-CoPaM co-expression point of view, adding stronger evidence to the findings of Gilmore and Tkach and their co-workers [12,13].

Furthermore, it was shown by Longhese *et al*. [24] that RPA interacts with both DNA polymerase α-primase complex and DNA polymerase δ complex. Bi-CoPaM finds that most of this complex's components are co-expressed with the RPA components as well as the CMR1 gene at very tight levels of the cluster C1. The significance of co-expression of DNA polymerases, CMR1 and related genes, and protein complexes is provided in §4.2.

### 4.2. DNA polymerases and the mini-chromosome maintenance complex (MCM2–7)

DNA polymerases catalyse DNA replication and repair. A striking level of co-expression among these complexes' genes is clear in our Bi-CoPaM results. All of the units composing DNA polymerase δ complex (POL3, POL31 and POL32), three of the four units composing DNA polymerase α complex (POL1, POL12 and PRI2), and two of the five units composing DNA polymerase *ɛ* complex (POL2 and DPB2) are found in the C1 cluster's core at the very strict level of tightness of DTB with **δ** = 0.98. Most of these genes also appear at **δ** = 0.99 and two of them (POL2 and POL3) appear at the absolute strictest case of IB. In fact, those units of DNA polymerase complexes that have not been included in C1 at these extreme levels of tightness are not present in the 500 genes considered in this study, indicating the 100 per cent clustering accuracy of Bi-CoPaM for the known components of the DNA polymerase complexes.

Moreover, the proliferating cell nuclear antigen PCNA/POL30, which functions as a sliding clamp for the DNA polymerase δ, is found in the cluster C1 at the very tight level of DTB with **δ** = 0.95. In Huttner & Ulrich [25], it was shown that the RPA complex is required for the ubiquitylation of PCNA at the replication fork. Again, Bi-CoPaM finds that all of these co-operating genes are tightly co-expressed.

It was shown that POL2, the catalytic unit of the DNA polymerase *ɛ*, interacts extensively with MRC1 at the G1/S checkpoint and during the S phase for DNA replication and in response to DNA damage on the leading strand [26]. Lou *et al.* [26] have also shown that MRC1 and POL2 co-immunoprecipitate and associate with CDC45, GINS and the MCMs. They then provided a model for the functional interaction between these proteins on the leading DNA strand during both normal DNA replication and replication stress. Interestingly, POL2 and CDC45 appear within C1 cluster at the tightest case of IB (figure 4), MRC1 and other genes associated with it as components of the replication checkpoint complex such as CSM3 and TOF1 [27] appear in C1 at the very tight case of DTB with **δ** = 0.99, whereas GINS is not one of the 500 genes included in this study.

The MCMs have a different story; Lou and co-workers proposed a parallel model at the lagging DNA strand during replication in which the six MCM units, MCM2, MCM3, MCM4, MCM5, MCM6 and MCM7, are loaded in late M phase and early G1 phase onto chromatin to form the ring-shaped heterohexamer complex MCM2–7 [26,28]. Then, the DNA polymerase *α* is loaded onto the chromatin and recruited to the MCM2–7 complex by MCM10, CTF4 and CDC45 [29].

The notable aspect of our results in relation to previous observations is that CDC45, most of the units in the DNA polymerase *α* complex, and CTF4 are found within the very tight cluster C1 at DTB with **δ** = 0.99 (except for MCM10 which is not one of the 500 genes considered in this study). More interestingly, the six MCM units within the MCM2–7 complex are all included in the same co-expressing comparatively tight cluster C3 at DTB with **δ** = 0.5 with five of them also included in the even tighter core case at **δ** = 0.6 (figure 4).

The genes in the cluster C3 show peak expression at the late M phase and the early G1 phase (table 2). The observations drawn from the two models at the leading and the lagging DNA strands during the replication checkpoint [26], in addition to the clear phase shift between the MCMs in the cluster C3 and the replication checkpoint genes in the cluster C1 shown in our tight co-expression clustering results, would indicate that the MCMs are co-regulated with the replication checkpoint genes, not only at the lagging strand but at the leading strand as well.

### 4.3. Yeast cohesion complex

The protein SMC3, which was found previously [12] to be associated with the four histones, is found within the tightest case of the cluster C1 in our study (figure 4). SMC3 associates with SMC1 to make a heterodimer that associates with MCD1 and IRR1 to compose the yeast cohesion complex, which is required for sister chromatid cohesion [30,31]. Interestingly, SMC1 and SMC3 are found in the tightest case of C1 at IB (figure 4), whereas MCD1 and IRR1 are found as well at DTB with **δ** = 0.99. Bi-CoPaM proposes that these genes are not only physically associated but also tightly co-expressed.

### 4.4. CMR1 as a potential target for the Mlu1 cell-cycle box binding factor complex

Many genes that are expressed at the G1/S transition have the Mlu1 cell-cycle box (MCB) element in their promoters [15,32,33], which is the target for the MCB binding factor (MBF) complex for transcription [22].

McIntosh [34] provided a list of 31 budding yeast genes that are regulated (or potentially regulated) by the MBF complex. Strikingly, 20 of these genes are included in our C1 cluster at no looser levels of tightness than DTB with **δ** = 0.95 except for the gene RFA3 that appears in C1 at MVB. Ten of these 31 genes are not included in our study, and one gene, TOP2, is included but clustered differently in C4 at the MVB case. The 20 matched genes are CDC21, RNR1, POL1, POL2, POL3, POL12, POL30, PRI1, DPB2, CTF4, CDC9, RFA1, RFA2, RFA3, RAD5, RAD51, TAD54, CLN1, CLN2 and PCL1. Four of these 20 genes survived up to the tightest case of IB. The cohesion complex genes SMC1, SMC3, MCD1 and IRR are among the genes that have the MCB element in their promoters [31]. This adds four more genes to the list of genes that have this element and appeared in the C1 cluster in our results. Moreover, SMC1 and SMC3 are among the 19 genes in the C1 cluster at IB.

The MBP1 protein, which associates with SWI6 to compose the MBF complex, is found in the core of the C4 cluster (table 3). As shown in table 2, the average peak time for the members of this cluster is between S and G2 phases that might indicate that the transcription of the G1/S transition genes starts at the middle of the previous cell cycle. According to the datasets used in this study (figures 2 and 3), the time period between the peaking of the MBP1 gene expression and the first following peaking of the G1/S transition genes is about 40 min.

The facts, that CMR1 has an MCB element in its promoter [22] and that Bi-CoPaM finds it tightly co-expressed with the G1/S transition genes, indicate strongly that the MBF complex is the transcription factor for the CMR1. Although previous studies have indicated the possibility of the CMR1 gene being a potential target for the MBF complex [22], it has not been confirmed yet; our results give stronger evidence to that especially by the survival of the CMR1's membership in the cluster that includes the G1/S genes even at the tightest case of IB.

### 4.5. Gene ontologies analysis

**Figure 5.**
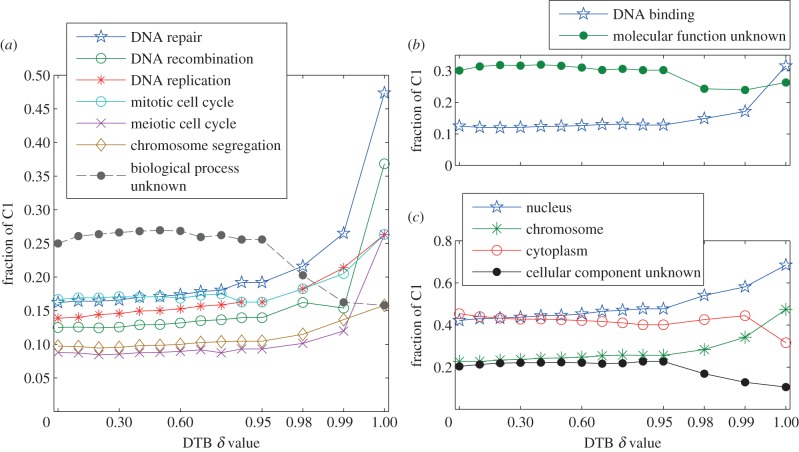
Summary of the GO Slim analysis for the cluster C1 at varying values of *δ*. The fraction of genes in C1 which represent the most significantly enriched GO terms for (*a*) processes, (*b*) functions and (*c*) components, are shown in this figure. Note that the *x*-axis, which shows the *δ* values, has been unfolded in the interval [0.9,1] because there are more details in this fine interval than the rest of *δ* values. (Online version in colour.)

At all of the adopted **δ** values, figure 5*a*–*c* show the fractions of C1 cluster genes that represent the most enriched GO terms for processes, functions and components, respectively. Figure 5*a* shows that DNA-related and cell-cycle-related processes have led the biological processes represented by the genes in C1. This quantitatively and systematically supports our analysis in the previous sections. This is even more strongly supported by the fact that the most enriched biological function in this cluster is DNA binding (figure 5*b*), and the most enriched cellular component is the nucleus (figure 5*c*). Indeed, DNA repair, replication and recombination in budding yeasts require DNA binding proteins and occur in the nucleus.

CMR1 has been annotated with the function ‘DNA binding’ and has been reported to localize in the nucleus as well as the cytoplasm, but its GO process term is still ‘biological process unknown’. The findings of our study, backed up with the finding by the studies of Gilmore, Choi and Tkach [11–13], help in directing the research towards unveiling the correct biological process for CMR1.

### 4.6. Other genes included in the C1 cluster core

RAD27 is a nuclease that participates in many DNA-related processes such as DNA replication, base excision repair and maintaining genome stability [35]. It was found that the cells with a deleted RAD27 are sensitive to the DNA damaging agent methyl methanesulphonate (MMS) and the ultraviolet (UV) light [36]. This information might link with the possible role of CMR1 in the UV DNA-damage response pathway [11]. RAD27 and CDC9 were found to be brought to the replication fork during the lagging DNA strand replication by the sliding clamp PCNA (POL30) [37]. Here, the relationship between RAD27 and the genes EXO1, RAD2 and RAD51 in DNA repair processes was proposed [37]. In our results, RAD27 and EXO1 are found within the tightest C1 cluster at IB, whereas PCNA (POL30), CDC9 and RAD51 are found in the same cluster at DTB with **δ** = 0.99.

Another gene included in the tightest cluster C1 at IB is MSH2 that forms a heterodimer with MSH6 to repair base-pair mismatches in DNA [38]. This gene MSH6 is also found in C1 at DTB with **δ** = 0.99.

An epistatic miniarray map has been constructed that shows quantitative measurements of the genetic interactions between 743 budding yeast genes from different chromosome biology pathways [2]. By clustering a subset of this array that have the DNA replication and repair genes based on their patterns of mutual genetic interactions (not genetic expressions), the genes RTT101, RTT107, MMS1, MMS22, RTT109 and ASF1 formed a small cluster which was investigated further [2]. It was found that RTT109 and ASF1 participate in the histone H3 K56 acetylation pathway at the transmission to the S phase. This was significant because the 25 gene products found to be associated with the four histones (figure 4) do not include any of these genes [12]. Nonetheless, in our tightly co-expressing clusters, the genes RTT107, RTT109 and ASF1 are found in the tight cluster C1 at DTB with **δ** = 0.98 (RTT107 was found at IB; figure 4).

We also note that the gene YRF1 is repeated seven times in the budding yeast genome and all of them appear in C1 at the very tight level of DTB with **δ** = 0.98.

### 4.7. Summary and conclusions

The new paradigm of clustering realized by the Bi-CoPaM method possesses the unique ability to perform tightness-tunable ensemble clustering, and it has been adopted to generate a small subset of tightly co-expressed genes. This subset and another subset generated by Gilmore *et al.* [12] are two different but overlapping small CMR1-including subsets of genes involved in DNA-related cellular processes such as DNA replication, repair and transcription. This complements the quantitative proteomics approach described by Gilmore *et al.* [12] extending the core histone network. The common factor for the genes in the subset provided in Gilmore *et al.* [12] is the association with the four histones, whereas the common factor for the genes in our results is highly synchronous co-expressions through the cell cycle.

The notable observation in both subsets is the existence of strongly functionally related genes that are often components of the same protein complex or the same pathway. The three components of the replication protein A (RPA); RFA1, RFA2 and RFA3 seem to be the closest to the newly characterized gene CMR1 in that RFA2 appeared in both sets of results associating with the four histones, associating with CMR1 and co-expressed with it, and that RFA1 and RFA3 appeared in the same subset of CMR1 in either results. Gilmore and co-workers [15] explored the relationship between CMR1 and the RNA polymerase complex III. Although they noticed the possibility that CMR1 would participate in the DNA repair at the G1/S checkpoint, they did not investigate this further. Our results suggest such a relationship may be functionally significant.

We propose that CMR1 may have a functional relationship not only with DNA polymerases but also with the cohesion complex. Most of the components of the DNA polymerases *α*, *δ* and *ɛ* are found to be tightly co-expressed with CMR1 and suggests a possible role of CMR1 in DNA replication and repair. SMC3, a core component of the cohesion complex, is found in Bi-CoPaM results and by Gilmore *et al.* [12] was associated with CMR1, whereas the other components of the complex were associated with CMR1 in our analysis. The strong association of CMR1 with the known targets of the MBF complex even in the extreme tightest cases clearly suggests the hypothesis that CMR1 expression is controlled by MBF complex and can be tested in future experimental work.

Taken together, our results have highlighted important subsets of genes based on the computational analysis of high-throughput data from different experiments instead of pure biological or biochemical experiments. They not only add stronger evidence for the main findings of the study of Gilmore *et al.* [12], but they also strongly highlight areas of less previous attention about the function of the CMR1 gene. CMR1 has been postulated to have functions in DNA processing. We have shown its expression through the cell cycle would support a relation between CMR1 with the RPA complex, DNA polymerases and the cohesion complex in addition to its role at the G1/S transition.

Finally, we also provide novel co-expressing genes clusters with tunable tightness levels. The evidence for the validity of these clusters' tight cores comes from genes that are strongly related by being in the same complex or pathway. These novel clusters can serve as an important resource for further focused gene discovery studies.
